# Survival among children under-five in India: a parametric multilevel survival approach

**DOI:** 10.1186/s12889-023-15138-4

**Published:** 2024-04-09

**Authors:** Ajit Kumar Jaiswal, Manoj Alagarajan, Wahengbam Bigyananda Meitei

**Affiliations:** 1 Mumbai, India; 2 Department of Fertility and Social demography, International Institute for Population Sciences, Mumbai, India; 3 Department of Public Health and Mortality Studies, International Institute for Population Sciences, Mumbai, India

**Keywords:** Under-five Mortality, Survival analysis, India, Parametric, Intra-class Correlation

## Abstract

**Background:**

Many studies have been conducted on under-five mortality in India and most of them focused on the associations between individual-level factors and under-five mortality risks. On the contrary, only a scarce number of literatures talked about contextual level effect on under-five mortality. Hence, it is very important to have thorough study of under-five mortality at various levels. This can be done by applying multilevel analysis, a method that assesses both fixed and random effects in a single model. The multilevel analysis allows extracting the influence of individual and community characteristics on under-five mortality. Hence, this study would contribute substantially in understanding the under-five mortality from a different perspective.

**Method:**

The study used data from the Demographic and Health Survey (DHS) acquired in India, i.e., the fourth round of National Family and Health Survey (2015–16). It is a nationally representative repeated cross-sectional data. Multilevel Parametric Survival Model (MPSM) was employed to assess the influence of contextual correlates on the outcome. The assumption behind this study is that ‘individuals’ (i.e., level-1) are nested within ‘districts’ (i.e., level-2), and districts are enclosed within ‘states’ (i.e., level-3). This suggests that people have varying health conditions, residing in dissimilar communities with different characteristics.

**Results:**

Highest under-five mortality i.e., 3.85% are happening among those women whose birth interval is less than two years. In case of parity, around 4% under-five mortality is among women with Third and above order parity. Further, findings from the full model is that ICC values of 1.17 and 0.65% are the correlation of the likelihood of having under-five mortality risk among people residing in the state and district communities, respectively. Besides, the risk of dying was increased alarmingly in the first year of life and slowly to aged 3 years and then it remains steady.

**Conclusion:**

This study has revealed that both aspects viz. individual and contextual effect of the community are necessary to address the importance variations in under-five mortality in India. In order to ensure substantial reduction in under-five mortality, findings of the study support some policy initiatives that involves the need to think beyond individual level effects and considering contextual characteristics.

## Background

The World Health Organization (WHO) defines under-five mortality as the death of a child before the age of five [1]. As an unfinished agenda of MDGs, reducing U5 mortality was included in the Sustainable Development Goals (SDGs) [2]. According to the United Nation Inter-Agency Group for child Mortality Estimation (UNIGME), global under-five mortality rate decreased to 39 deaths per 1000 live births in 2018 from 93 in 1990 and 76 in 2000. Around 59% decline was observed in total number of U-5 deaths in 2018 (i.e., 5.3 million) from 12.5 million in 1990 [3].

Despite a significant reduction in global under-five mortality rates, from 90.6 per 1000 live births in 1990 to 42.5 per 1000 live births in 2015 [4] an estimated 5.3 million children under the age of five died each year [5].

The burden of under-five mortality is unevenly distributed, with a strong concentration in middle- and low-income countries [6, 7]. In India, under-five mortality is an important health problem and has been declining steadily. According to National Family Health survey, total 109 death per 1000 live births were taking place within the initial 59 months of life in 1992–93 [8]; around 95 in 1998–99 [9]; and 75 in 2005–06 [10]; and 50 in 2015–16 [11]. As per Sample Registration System 2015 estimates, under-five mortality rate in India varied from 13 to 62 deaths per 1,000 live births across states [12].

Many studies have been conducted on under-five mortality in developing countries like India and most of them focused on the associations between individual-level factors and under-five mortality risks. But, only a scarce number of literatures talked about contextual level effect on under-five mortality. For instance, studies examined that factors such as low level of education, unimproved drinking water and sanitation, low family income, short birth interval, and birth delivery place continue to put children at risk [13–16]. Besides, there are some demographic determinants associated with under five mortality such as maternal age at birth, birth spacing pattern, parity, and size of the children at birth [17, 18]. Studies show that infant and child mortality is high among the first born, but relatively low among second and the third order births [19]. The length of the birth interval has a negative association with the infant and child mortality, i.e., the smaller the birth interval, the higher is the child mortality [20]. A notable increase in the coverage of interventions relevant to child survival, such as births in a health facility, skilled birth attendance, antenatal care visit, coverage of breastfeeding within 1 h of birth and exclusive breastfeeding for children etc. have a significant contribution in reducing child mortality [21].

A great variations in the availability of maternal and child healthcare services are observed among the communities. These community settings are important aspects and may also be relevant in exacerbating or mitigating inequities of population health outcomes across regions [22, 23]. Incorporating community-level factors into under-five mortality analyses allows for the identification of health risks associated with specific social structures and community ecologies, which is a critical policy tool for the development of public health interventions [24, 25]. The contextual phenomenon is the inherent opinion of social epidemiology; examining people form residing in the same locality, it resembles that people are almost the same in terms of their health outcomes. Thus, in any population, it is crucial to understand the individual’s health outcome without considering contextual factors, either at design or in analyses. In developed and developing countries, under-five mortality is yet to be studied at multiple levels.

Hence, it is very important to have thorough study of under-five mortality at various levels. This can be done by applying multilevel analysis, a method that assesses both fixed and random effects in a single model. The multilevel analysis allows extracting the influence of individual and community characteristics on outcome. In contrast, the application of individual analyses makes it difficult to understand whether variation at the community level is due to their characteristics in the absence of community-level factors. Hence, this study would contribute substantially in understanding the under-five mortality from a different perspective.

## Method

### Data source

The study used data from the Demographic and Health Survey (DHS) acquired in India, i.e., the National Family and Health Survey (2015–16). It is a nationally representative repeated cross-sectional data. The main objective of NFHS-4 is to obtain reliable, valid and comparable data on levels of health and family welfare across a range of key domains for reproductive populations. The survey provides information on a number of indicators included in the Sustainable Development Goals (SDGs) which India is committed to.

Mortality data for the children under a particular mother can be obtained from the complete birth history file provided in the survey data. In the current analysis, we considered only those women who had given most recent births in the 5-years preceding to the survey. Hence, data of 1,90,898 cases were extracted from the Birth file database (i.e., IABR71FL).

### Variable description

#### Outcome variable

The outcome variable for this study is the risks of death in childhood, measured as the duration of survival since birth in months. It is defined as the risk of death of the child from birth to 59 months (i.e., under-five mortality). Analysis in this study is restricted to live births in the last five years prior to the survey. The children’s survival status and the age at death in months (if the child had died) or the last month they were known to be alive (if the child was still living at the time of the survey) were combined to generate the outcome variables for the survival analysis. Those children who have died before completing 59 months of their lives are considered as ‘cases’ and they were non-censored, whereas children who were still alive at the time of the survey were treated as right-censored.

#### Explanatory variables

Individual and community level variables that were considered for viable associations with childhood mortality were based on a set of earlier studies [26–29]. Taking into account of the available information in NFHS-4, a set of various socio-economic, demographic variables along with health services utilization factors are obtained in this study. In addition, to examine the community level impact on under-five mortality, proportion of women assessed Full ANC, Institutional delivery and Postnatal Care was generated at district level, in which the participants were dwelling. A couple of studies have utilized community socioeconomic disadvantage as a community-level determinant [30–33]. We have included available variables pertaining to various policies and programs already in progress to validate the robustness of the estimates and state causation.

### Statistical analysis

Multistage sampling-based this DHS data encompasses respondents by different geographic areas, and the respondents within the same geographical areas are more prone to be correlated with each other. Descriptive analysis was done by evaluating the prevalence of the dependent variable i.e., under-five mortality across the categories of many independent variables.

Multilevel Parametric Survival Model (MPSM) was employed to assess the influence of contextual correlates on the outcome. There are two reason why we employ MPSM in the study. First, this model is appropriate in analysing censored observations. The second reason for using the model is to account for the hierarchical structure of the NFHS data.

The assumption behind this study is that ‘individuals’ (i.e., level-1) are nested within ‘districts’ (i.e., level-2), and districts are enclosed within ‘states’ (i.e., level-3), [34]. This suggests that people have varying health conditions, residing in dissimilar communities with different characteristics. We separately analysed and assessed the association between under-five mortality and Community level characteristics to examine the extent to which the covariates at community level influence under-five mortality in India.

## Results

Table 1 lists the background characteristics of the sample population. The majority of the sample population was resided in rural areas (75%), had a secondary education (46.6%). Around 24.5% of population belongs to poorest wealth quintile and only (15%) belongs to richest quintile group. Further, around only (5.7%) of population resided in Pakka type of house and more than (9%) of population resided in Kachcha type of houses.

**Table 1:** Background characteristics of the sample population

Covariates	Child Mortality rate
**Place of Residence**	
Urban	47,833 (25%)
Rural	143,065 (75%)
**Birth Interval**	
Less than 2 years	35,402 (27.5%)
2–3 years	38,431 (29.9%)
More than 3 years	54,662 (42.5%)
**Parity**	
First–Second	124,291 (65.1%)
Third and above	66,607 (34.9%)
**Educational level**	
No education	55,165 (28.9%)
Primary	26,712 (13.9%)
Secondary	88,871 (46.6%)
Higher	20,150 (10.6%)
**Wealth Index**	
Poorest	46,782 (24.5%)
Poorer	43,739 (22.9%)
Middle	38,393 (20.1%)
Richer	33,212 (17.4%)
Richest	28,772 (15%)
**Quality of water**	
Improved	156,485 (82%)
Unimproved	24,121 (12.6%)
Others	10,292 (5.4%)
**Stool disposal of the child**	
Safe	67,198 (36.2%)
Unsafe	118,231 (63.8%)
**Toilet Facility**	
Improved*Not shared	82,834 (44.8%)
Shared	16,679 (9%)
Unimproved	4,276 (2.3%)
No facility	80,780 (43.7%)
Others	287 (0.2%)
**Cooking Fule**	
Clean	62,123 (32.5%)
Solid	128,757 (67.4%)
**Type of house**	
Kachcha	17,587 (9.2%)
Semi Pakka	162,401 (85.1%)
Pakka	10,910 (5.7%)
**Total**	190,898 (100%)

**Fig. 1:**
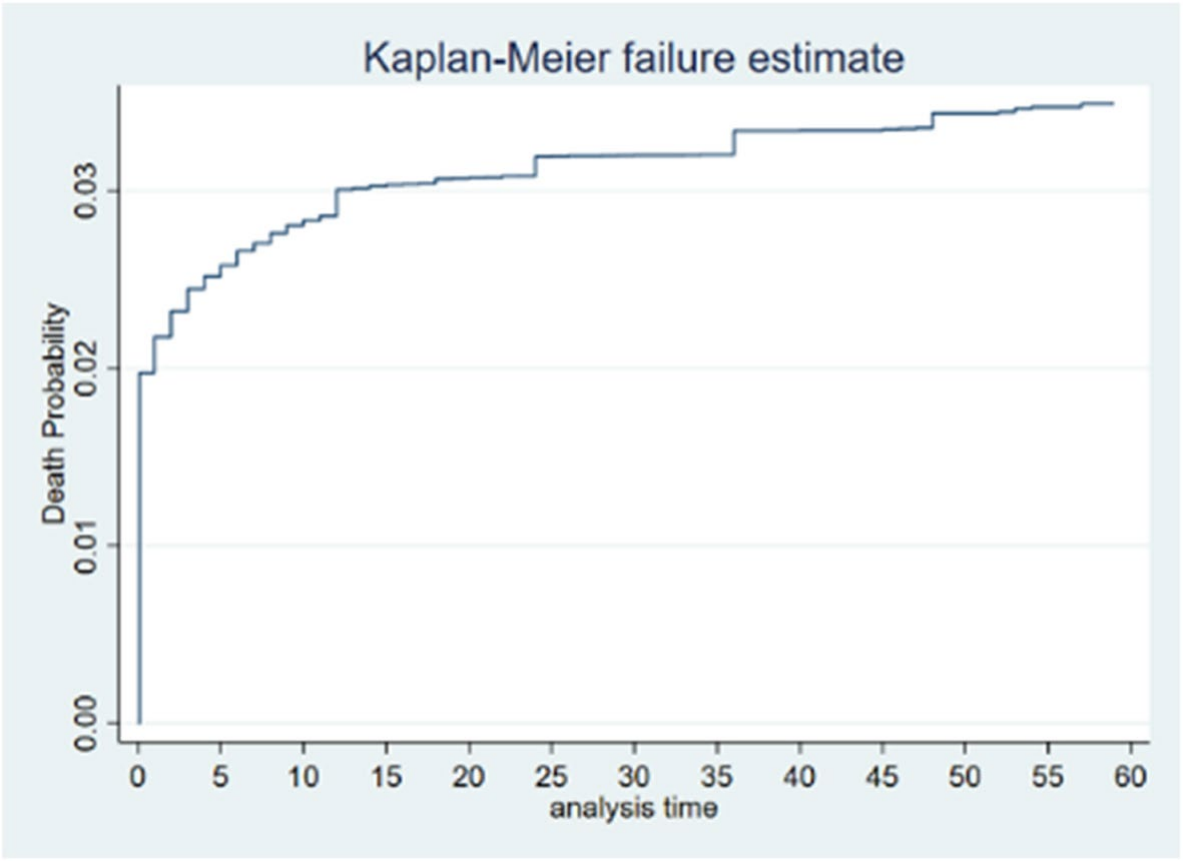
The overall Kaplan–Meier curve of the survival status of under five mortality in India

The Kaplan–Meier failure curve indicated that the probability of under-five mortality increased over time. The risk of dying was increased alarmingly in the first year of life and slowly to aged 3 years and then it remains steady (Fig. 1).

**Fig. 2:**
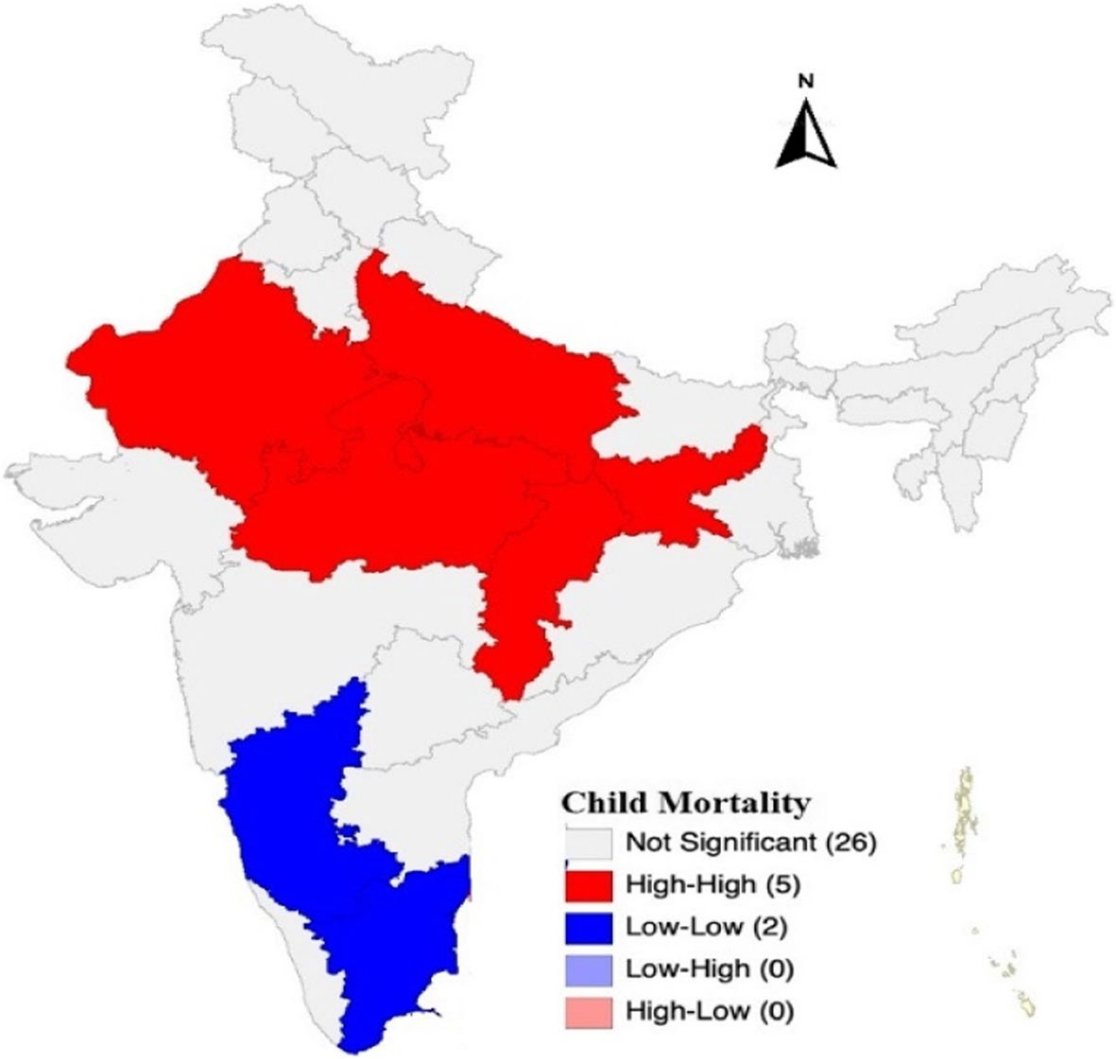
Univariate LISA cluster map of child mortality at state level of India

Figure 2 presents the spatial variation in under five mortality in Indian states. The univariate LISA cluster map shows the states in 5 categories, those are not significant, high surrounded by high, low surrounded by low, low surrounded by high and high surrounded by low. We can see in the map that there are 5 sates (Uttar Pradesh, Madhya Pradesh, Rajasthan, Chhattisgarh, Jharkhand) which are shaded red, have high prevalence of under 5 child mortality which is effected by the high prevalence of neighbouring states. In south we can see, Tamil Nadu and Karnataka are shaded blue, that represents those states have low prevalence of under 5 child mortality which is effected by low prevalence of neighbouring states. There is no state which is under low surrounded by high or high surrounded by low. The rest of the states are not significant.

**Fig. 3:**
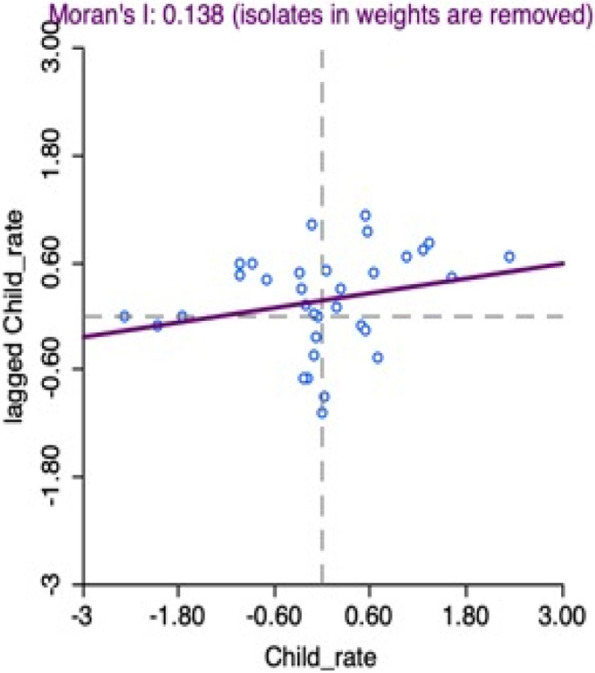
Moran’s I for spatial autocorrelation of child mortality of India

The scatter Diagram gives the scatter plot of the variable in x axis with lagged (values of neighbouring states) in y axis. Global Moran’s I is also given. From this diagram we can see that global Moran’s I for child mortality of India is 0.138, which reflects the under 5 child mortality of states of India is moderately correlated and positively affected by the neighbouring states (Fig. 3).

**Fig. 4:**
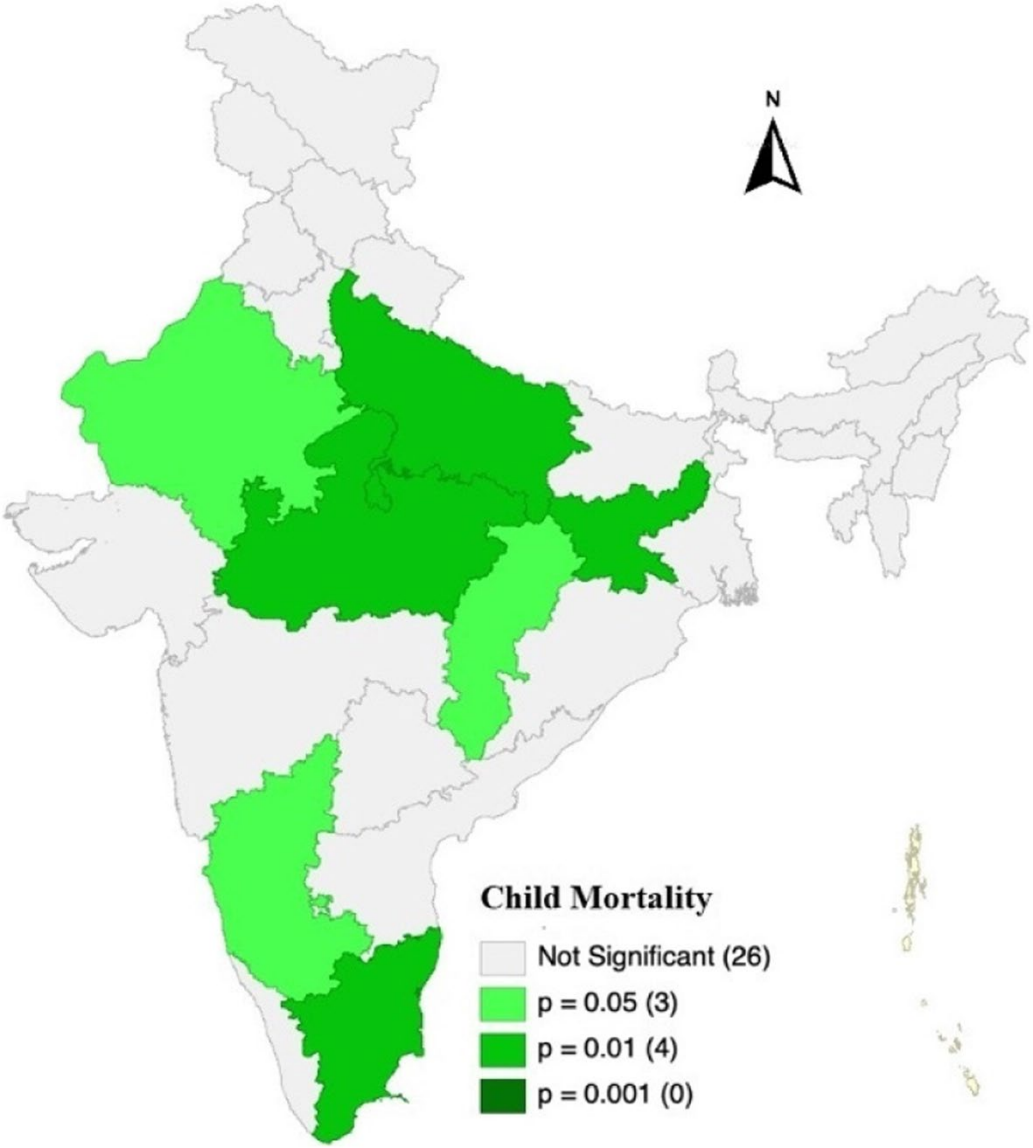
Significance map of child mortality at state level of India

Figure 4, the map shows the states which are significantly spatially distributed with various shades of green. Darker the green shade we can say with higher precision that it is spatially distributed. From this map we can interpret that under 5 child mortality of four states (Uttar Pradesh, Madhya Pradesh, Jharkhand, Karnataka) are moderately significant (*p* < 0.01) that those are affected by the prevalence of under 5 child mortality of neighbouring states. Lighter shaded states are also affected (*p* < 0.05) by surrounding state’s under 5 child mortality. Grey shaded states are not significant that those are affected by their neighbouring states. There is no state which is highly significant that is affected by neighbouring state.

Table 2 shows the percentage of under-five mortality by Socio-economic characteristics. About 3.24% under-five mortality are taking place in children residing in rural areas than its counterpart where prevalence is only 1.96%. Highest under-five mortality i.e., 3.85% are happening among those women whose birth interval is less than two years. In case of parity, around 4% under-five mortality is among women with Third and above order parity. Children of the illiterate and poorest women have highest death prevalence. For instance, 4.39 and 4.36% is the prevalence of under-five mortality among the illiterate and poorest wealth quintile women, which is also the highest. It is observed that households using High polluting fuels are having 3.45% of U-5 mortality which is more than double of the houses using low polluting fuels. Similarly, from the Kachcha houses, 3.44% child deaths are taking place; while from Pakka houses, it is 2.45%.

**Table 2:** Prevalence of Under-five mortality rate per 1000 live births by some Socio-economic characteristics with 95% CI in India, 2015–16

Covariates	Child Mortality rate
**Place of Residence**	
Urban	2.39 (2.11, 2.67))
Rural	3.93 (3.80, 4.07)
**Birth Interval**	
Less than 2 years	4.52 (4.18, 4.85)
2–3 years	3.42 (3.09, 3.76))
More than 3 years	3.18 (2.94, 3.41))
**Parity**	
First–Second	2.68 (2.51, 2.84))
Third and above	5.00 (4.65, 5.36)
**Educational level**	
No education	5.39 (5.00, 5.79)
Primary	4.05 (3.64, 4.46)
Secondary	2.65 (2.40, 2.90)
Higher	1.48 (1.29, 1.68)
**Wealth Index**	
Poorest	5.39 (5.07, 5.71)
Poorer	4.26 (3.87, 4.66)
Middle	3.36 (3.03, 3.70)
Richer	2.25 (1.89, 2.62)
Richest	1.43 (1.23, 1.67)
**Quality of water**	
Improved	3.49 (3.31, 3.67)
Unimproved	3.23 (2.83, 3.63)
Others	2.96 (0.63, 5.29)
**Stool disposal of the child**	
Safe	0.83 (0.75, 0.90)
Unsafe	1.20 (1.13, 1.28)
**Toilet Facility**	
Improved*Not shared	2.35 (2.13, 2.57)
Shared	3.13 (2.47, 3.78)
Unimproved	3.17 (2.09, 4.24)
No facility	4.68 (4.42, 4.94)
Others	3.04 (-0.07, 6.85)
**Cooking Fule**	
Clean	2.32 (2.09, 2.54)
Solid	4.18 (4.01, 4.35)
**Type of house**	
Kachcha	2.66 (2.09, 3.22)
Semi Pakka	3.55 (3.39, 3.71)
Pakka	3.42 (2.97, 3.87)
**Total**	34.6 (3.30, 3.62)

Table 3 presents the results of the extent to which Individual determinants along with community contextual factors influence variation in under-five mortality. Total, four models are employed in this study. The results of the null model which has no explanatory variable (i.e., Model 0) showed a significant variation in child mortality at individual and community levels. This also implies that the correlation between mothers living in the state and district communities regarding the likelihood of experiencing under-five mortality is 4.76 and 1.97%, respectively.

**Table 3:** Multilevel parametric survival model for under-five child mortality

	**Model 0**				**Model 1**				**Model 2**				**Model 3**			
	**HR**	***P*-value**	**CI**		**HR**	***P*-value**	**CI**		**HR**	***P*-value**	**CI**		**HR**	***P*-value**	**CI**	
**Constant**	0.0009	0.000	0.0008	0.0010	0.0022	0.000	0.0017	0.0028	0.0007	0.000	0.0005	0.0010	0.0010	0.000	0.0006	0.0015
**District level Variables**																
Prevalance of Full Ante-natal Care					0.42	0.000	0.27	0.65					0.38	0.004	0.19	0.73
Prevalance of Place Of Delivery					0.40	0.000	0.28	0.56					0.65	0.099	0.39	1.08
Prevalance of Full Post-natal Care					1.20	0.322	0.84	1.70					1.51	0.110	0.91	2.48
**Individual level Variables**																
**Place of Residence**																
Urban **®**																
Rural									0.94	0.376	0.82	1.08	0.92	0.252	0.80	1.06
**Birth Interval**																
Less than 2 years®																
2–3 yrs									0.64	0.000	0.58	0.71	0.64	0.000	0.58	0.71
More than 3 years									0.24	0.000	0.21	0.27	0.24	0.000	0.21	0.27
**Parity**																
First–Second®																
Third and above									1.48	0.000	1.33	1.64	1.47	0.000	1.32	1.63
**Educational level**																
No education®																
Primary									1.04	0.554	0.91	1.19	1.04	0.540	0.91	1.19
Secondary									0.87	0.031	0.77	0.99	0.88	0.039	0.77	0.99
Higher									0.77	0.066	0.58	1.02	0.77	0.069	0.58	1.02
**Wealth Index**																
Poorest®																
Poorer									1.01	0.820	0.89	1.15	1.03	0.636	0.91	1.17
Middle									0.92	0.340	0.78	1.09	0.96	0.593	0.81	1.13
Richer									0.90	0.327	0.72	1.11	0.94	0.596	0.76	1.17
Richest									0.69	0.012	0.52	0.92	0.73	0.036	0.55	0.98
**Source of water**																
Improved®																
Uimproved									0.92	0.326	0.79	1.08	0.91	0.244	0.78	1.07
Others									1.06	0.926	0.34	3.28	1.07	0.905	0.34	3.33
**Stool disposal of the child**																
Safe®																
Unsafe									0.94	0.328	0.83	1.06	0.93	0.281	0.83	1.06
**Toilet Facility**																
Improved*Not shared®																
Shared									1.05	0.601	0.87	1.26	1.06	0.566	0.88	1.27
Unimproved									1.14	0.446	0.81	1.61	1.16	0.393	0.83	1.63
No facility									1.14	0.085	0.98	1.32	1.17	0.040	1.01	1.35
Others									0.37	0.319	0.05	2.63	0.38	0.328	0.05	2.68
**Cooking Fule**																
Clean®																
Solid									1.10	0.238	0.94	1.29	1.09	0.287	0.93	1.28
**Type of house**																
Kachcha®																
Semi pakka									0.97	0.737	0.81	1.16	0.96	0.648	0.80	1.15
Pakka									1.27	0.088	0.96	1.67	1.26	0.099	0.96	1.66
**Variance (_cons) [State]**	0.16		0.09	0.30	0.10		0.05	0.19	0.06		0.03	0.14	0.04		0.02	0.10
**ICC (%)**	**4.76**				**2.91**				**1.90**				**1.17**			
**variance (_cons) [State > District]**	0.07		0.05	0.09	0.04		0.03	0.07	0.03		0.01	0.10	0.02		0.00	0.10
**ICC (%)**	**1.97**				**1.29**				**0.86**				**0.65**			

After incorporating only contextual factors into the models-1, risks of death remained significant but slightly plummeted in all regions. Prevalence of ANC, institutional delivery and PNC at district level are integrated and it is found that districts with high proportion of women who are assessing full ANC and institutional delivery are facing about [58%; *p* < 0.001; CI = 0.27–0.65] and [60%; *p* < 0.001; CI = 0.28–0.56] less hazard of under-five mortality, respectively. Moreover, as the district level variables taken into account, it is found that and the particular values of the variance at state and district levels (i.e. ICC = 2.91 and 1.29%) from the models explains total variance in under-five mortality can be assigned among people living in the same communities.

Further, Model 3 is applied considering only individual characteristics and it is noticed that children from richest wealth indexed houses has a lesser [31%; *p* < 0.01; CI = 0.52–0.92] chances of dying before attaining the age of 5 years. Households without toilet facilities has higher [14%; *p* < 0.01; CI = 0.98–1.32] likelihood of U-5 mortality. The measures of variation remained significant across communities, ICC associated with chances of U-5 mortality estimated as 1.90 and 0.86% across state and district levels, respectively.

Further, full model (i.e. model 4) is conducted, which includes all the selected individual and community level variables. Women who practiced long birth spacing such as birth interval with *2–3 years* and *More than 3 years* are having less hazard ratio with [36%; *p* < 0.001; CI = 0.58–0.71] and [28%; *p* < 0.001; CI = 0.211–0.27], respectively of under-five mortality than the women with less than 2 years of birth interval. Children from the Women of third and above parity are around [47%; *p* < 0.001; CI = 1.32–1.63] more likely to bear the risk of mortality than the women of up to second parity. As the educational status of the mother increased to secondary and higher level, chances of under-five mortality declined by 12 and 23% (*p* < 0.001), respectively. Findings reveal from the full model (i.e., Model 2) that ICC values of 1.17 and 0.65% are the correlation of the likelihood of having under-five mortality risk among people residing in the state and district communities, respectively.

## Discussion

The focus of this paper was to examine the individual and community level factors’ effect on under-five mortality and determine the extent to which regional variation affect Under-five mortality in India. There are many studies that have concluded that the risk of death during childhood are because of combined effects of contextual and individual variables, and the effects of exogenous factors at those levels [31, 35, 36]. Whitworth & Stephenson (2002) also found that village- or community-level factors were more important in accounting for child mortality than mother- or individual-level characteristics [37].

Our descriptive findings reveal that U-5 mortality is quite higher in rural areas than urban areas. There might be a couple of reasons behind the phenomena. First, three fourth of the respondents were from rural areas, whereas only one-fourth from urban areas. This huge differential between rural–urban population structures across regions seems to contribute to regional deaths differential in India. Second, it is obvious that the differentials in the distribution of health care facilities exist between rural and urban communities [38, 39].

It is also established that many of the characteristics at the individual and community levels considered in this study were found to be significantly associated to under-five mortality. Lack of proper sanitation is a major public health risk that affects child health much more than other members of the communities. Child mortality is majorly affected by household hygiene. Many of the households still do not have access to modern toilet facilities particularly communities living in the hills [40].

In our study, apart from individual characteristics, the community level characteristics, i.e. full ANC and delivery in health care facility, exert significant effect on under-five mortality includes full ANC and delivery in health care facility. This finding may be attributed to inequality in the distribution and use of health facilities [38, 39, 41, 42].

After adjusting for the selected district-level and individual-level variables, finding suggested higher under-five mortality clustering are at the community level rather than individual level. For this, there may be a reasonable understanding that children’s interaction with community environment is more likely to be compared with the period under age one [35].

Health interventions targeting child mortality reduction require a prominent policy in order to achieve further reductions in infant mortality, and neo-natal mortality in particular. It is important to recognize that both preventive neonatal health care and treatment of various illnesses of neonates such as septicaemia, meningitis and pneumonia, which are referred to as sepsis, involves specialized skills for community health workers for home-based neonatal health care interventions. And the fact remains that hospital-based delivery by the skilled attendant and neo-natal and post-natal health care services, immediate breastfeeding and nutrition require attention.

## Conclusion

This study has revealed that both aspects viz. individual and contextual effect of the community are necessary to address the importance variations in under-five mortality in India. In order to ensure substantial reduction in under-five mortality, findings of the study support some policy initiatives that involves the need to think beyond individual level effects and considering contextual characteristics. Policies that address regional disparities in under-five mortality in India must include strategies to improve child health outcomes, particularly during the first year of life.

### Limitations and strengths

Besides, there are some caveats which is necessary to be addressed. First, the cross-sectional nature of the Data does not allow making cause and effecting relationship. Second, this study uses State and District-level as proxy for community, so this attribute of the data may lead to some information biasness because of misclassification of the respondents from different clusters [43]. Lastly, there might be problem of auto-correlation because some variables have been generated at Community level with individual-level variables. Further, few other important contextual factors such as cultural practices, customs, information on the quality of preventive and clinical services could not be addressed in this study, as they are not available in the utilized dataset. The study has its strength also, which need to be discuss here. Nonetheless, use of DHS dataset has its own strength. DHS datasets are nationally representative and one could easily generalize findings across the whole country. Also, the study looked into the influencing factors’ impact on under five mortality on various community levels.

## Data Availability

The datasets used in the current study are freely available from the Demographic Health Survey. https://dhsprogram.com/Data/.
